# CABeRNET: a Cytoscape app for augmented Boolean models of gene regulatory NETworks

**DOI:** 10.1186/s12859-016-0914-z

**Published:** 2016-02-04

**Authors:** Andrea Paroni, Alex Graudenzi, Giulio Caravagna, Chiara Damiani, Giancarlo Mauri, Marco Antoniotti

**Affiliations:** Department of Informatics, Systems and Communication, University of Milan-Bicocca, Viale Sarca 336, Milan, I-20126 Italy; Institute of Molecular Bioimaging and Physiology of the Italian National Research Council (IBFM-CNR), Via F.lli Cervi, 93, Segrate, I-20090 (MI) Italy; School of Informatics, University of Edinburgh, 10 Crichton St, Edinburgh, EH8 9AB UK; SYSBIO Centre of Systems Biology, Piazza della Scienza 2, 20126 Milano, Italy, Viale Sarca 336, Milan, I-20126 Italy; Milan Center for Neuroscience, University of Milan-Bicocca, Milan, Italy

**Keywords:** Gene regulatory networks, Cell differentiation, Attractors, Cancer development, Network augmentation, Robustness analysis

## Abstract

**Background:**

Dynamical models of gene regulatory networks (GRNs) are highly effective in describing complex biological phenomena and processes, such as cell differentiation and cancer development. Yet, the topological and functional characterization of real GRNs is often still partial and an exhaustive picture of their functioning is missing.

**Results:**

We here introduce CABeRNET, a Cytoscape app for the generation, simulation and analysis of Boolean models of GRNs, specifically focused on their augmentation when a only partial topological and functional characterization of the network is available. By generating large ensembles of networks in which user-defined entities and relations are added to the original core, CABeRNET allows to formulate hypotheses on the missing portions of real networks, as well to investigate their generic properties, in the spirit of complexity science.

**Conclusions:**

CABeRNET offers a series of innovative simulation and modeling functions and tools, including (but not being limited to) the dynamical characterization of the gene activation patterns ruling cell types and differentiation fates, and sophisticated robustness assessments, as in the case of gene knockouts. The integration within the widely used Cytoscape framework for the visualization and analysis of biological networks, makes CABeRNET a new essential instrument for both the bioinformatician and the computational biologist, as well as a computational support for the experimentalist. An example application concerning the analysis of an augmented T-helper cell GRN is provided.

## Background

Consistently with the increasing availability of *big data* regarding biological systems, is the need of *mathematical* and *computational models* aimed at their effective analysis and interpretation [1, 2]. Many methodologies aim at *inferring such models from the data*, with the final goal of selecting a unique descriptive model for a phenomenon of interest. Other techiques, instead, explore the space of all possible models via a *generative approach*, with the aim of *identifying the common characteristics*, properties and regularities of those models that are “consistent” with the data. The rationale underlying the latter approach is that, although lack of reliable data might prevent us to infer the exact true model, the statistical analysis of *ensembles* of *plausible* models can provide fundamental insights about the reference phenomenon, its origin and, in specific cases, its evolutionary history. This methodology is typical of *complex systems science*, which borrows ideas and techniques from *statistical physics*, in order to focus on the *emergent* dynamical behaviours and the so-called *generic* or *universal* properties of real-life phenomena, often by means of simplified mathematical and computational models [3, 4].

Within this context, one of the best examples, involving genomic data, is provided by Boolean models of *gene regulatory networks* (GRNs), which have repeatedly proved fruitful in describing key properties of real systems, as well as in providing cues and hints for wet-lab experiments (see, e.g., [3, 5–12]). The simulation of partially characterized regulatory architectures with a Boolean approach, in particular, has recently gained attention (see, e.g., [13–21]). A first motivation lies in the inherently “dynamical” nature of gene (de)regulation processes, and in the clear limitations of a “static” analysis capturing only a partial picture of such complex processes. For example, a structural analysis of the genomic interactions might preclude to determine the influence of a *target-selective therapy* on the overall GRN interplay ruling *tumorigenesis*. Moreover, as compared to other quantitative approaches, such as ODEs models (see [22] for a review on GRN modeling), the Boolean abstraction allows for a clear and effective characterization of the gene activation patterns (or *attractors* in the terminology of dynamical systems) characterizing the different phenotypic functions, such as cell types, modes and fates, under the metaphor of “emergent collective behaviours” [3, 23–31]. In this context, phenomena such as tumorigenesis can be explained as rare emergent pattern, triggered by *signals*, stochastic fluctuations and *biological noise* (see, e.g., [32, 33]).

In line with this approach, we here continue on a recent research strand that has involved the development of simulation and analysis tools for dynamical Boolean GRNs (see, e.g., [34–36]). The foundations of our theoretical framework lay in the seminal work by Stuart Kauffman on *Random Boolean Networks* (RBNs) [37] and, more recently, on the dynamical model of cell differentiation introduced in [38, 39] and based on *Noisy Random Boolean Networks* (NRBNs). In this theoretical framework, cell types are associated to dynamical gene activation patterns and differentiation to cell-specific noise-resistance mechanisms, the underlying hypothesis being that such mechanisms refine along with differentiation stages (see [40] and references therein). In the Background Section the main features of this approach are outlined.

In this paper we introduce CABERNET, a Cytoscape [41] app to generate, import, simulate and analyse Boolean models of GRNs. In addition to the simulation of specific regulatory architectures, a key feature of this new tool lies in the possibility of generating and analysing ensembles of GRN models sharing user-defined structural and dynamical features (such as, for instance, a specific degree distribution, a set of plausible regulatory functions, or a particular differentiation scheme), in order to investigate the generic properties of classes of GRNs, in the spirit of the previously mentioned ensemble-based generative approach. Most importantly, CABERNET allows to *augment* partially-characterized GRNs by adding user-defined entities and relations. The underlying motivation is that, despite the increasing knowledge on gene regulation in real organisms, the topological and functional characterization of real networks is stil far from being comprehensive, thus preventing to capture the complexity of the overall interplay. CABERNET is conceived to randomly generate the missing portions of these partially-characterized GRNs, in order to accommodate the advantages of the ensemble when we want to study GRNs that share a common core and other structural and functional parameters. Clearly, this shall allow to test hypotheses on the yet unknown missing portions of real networks.

In addition, CABERNET guides the user through various robustness analyses of the GRNs, which can be matched against genomic experimental data. Notice that CABERNET can also generate completely random GRNs with defined structural and functional constraints, as well as simulate the dynamics of completely characterized GRNs.

The paper is organized as follows. In the Background Section a quick overview of the GRN dynamical model is presented. In the Implementation Section the main features and functionalities of CABERNET are described. As a proof of principle, in the Results Section we present the augmentation of the T-helper cell signaling network and describe the analysis of its dynamics, with particular focus on the emergent differentiation scheme and its robustness against the knockout of specific genes. Conclusions are presented in the last Section.

## Background: the GRN model

In CABERNET, GRNs are represented as Noisy Random Boolean Networks (NRBNs) [39] (for an extended description of the model and a recent review on Boolean modeling please refer to [42]). Canonical RBNs are oriented graphs in which Boolean nodes represent (either active or inactive) genes, and edges stand for regulatory interactions, modeled as *boolean functions*. The dynamics of RBNs is synchronous and deterministic, and the activation value of each node is updated at every discrete time step, according to a node-specific Boolean function depending on the value of the input nodes. Accordingly, the overall dynamics eventually ends up in (at least unitary) state cycles, termed *attractors*, which represent the emerging gene activation patterns displayed by the GRN^1^

In the more recent NRBN model [39], transitions among attractors are made possible as a consequence of random modifications of the node’s activation value, lasting a defined time span (i.e., *flips*). Thus, it is possible to determine the so-called *Attractors Transition Network* (ATN, or *matrix*, ATM), i.e., a *stability matrix* displaying the noise-induced transition probability among attractors.

**Fig. 1:**
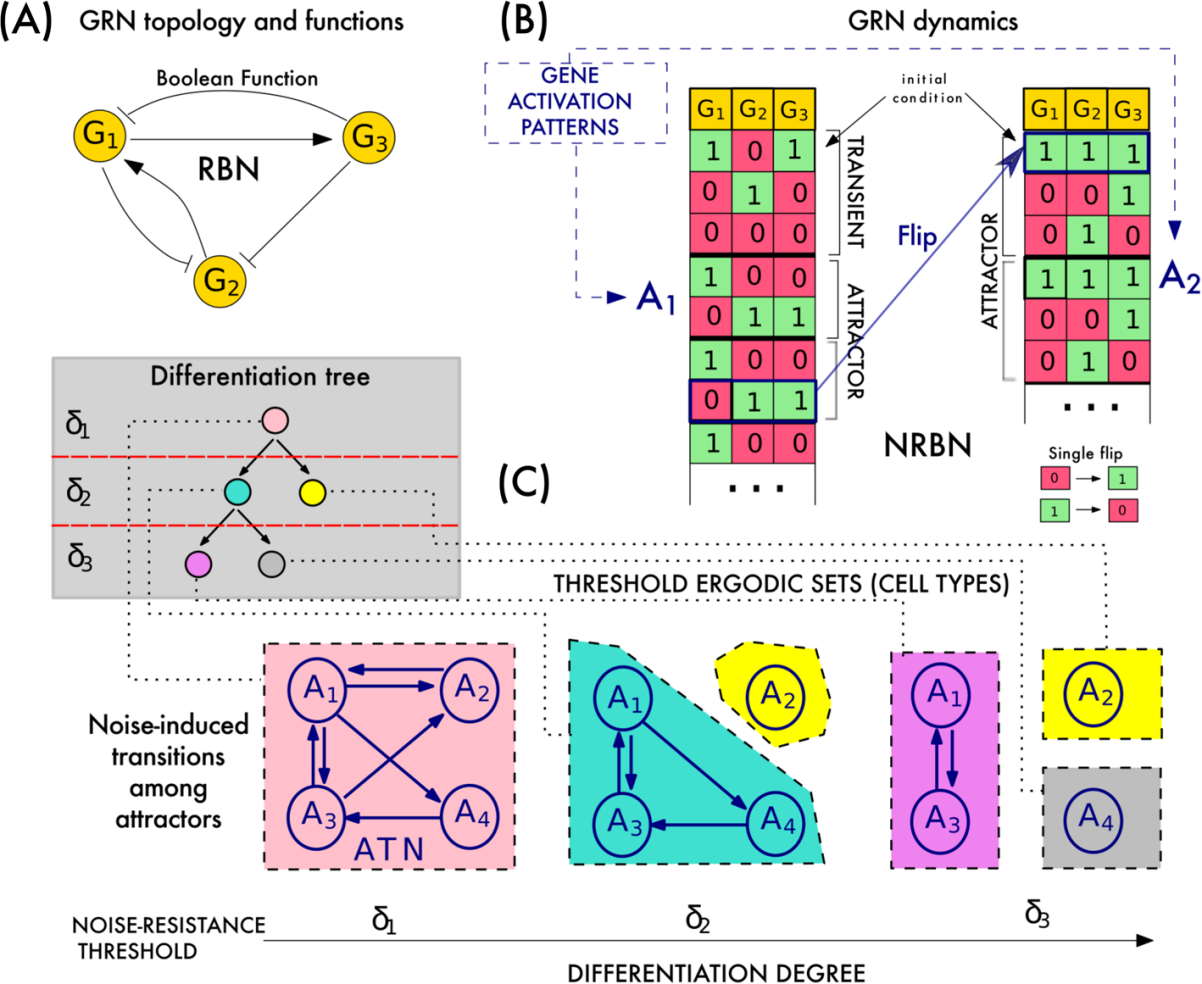
Simplified representation of the GRN model in CABERNET. **a** Example RBN with 3 genes (nodes) and edges representing regulatory interactions (via node-specific Boolean functions, not shown). **b** Example dynamics to highlight network’s attractors, modeling gene activation patterns *A*_1_, …, *A*_4_, and possible transitions among them induced by noise (i.e., single flips). **c** The transitions yield an Attractor Transition Network that generates 5 cellular types when 3 thresholds, *δ*_*i*_, *i*=1,2,3 are evaluated to asses the corresponding Threshold Ergodic Sets. In this approach, where the efficiency of noise-control mechanisms is related to differentiation types, stem cells (pink), intermediate stages (light blue) and fully differentiated cells (yellow, purple and grey) emerge. The corresponding differentiation tree, is shown (Fig. modified from [35])

In the biological metaphor proposed in [39], the sets of attractors in which the dynamics can wander via noise-induced transitions (also defined as *Threshold Ergodic Sets*, TESs) represent the gene patterns of specific cell types. Besides, given that it is hypothesized that less differentiated cell types are characterized by less refined noise-control mechanisms (see, e.g., [43–46]), different thresholds are introduced to exclude those transitions that are unlikely to occur for a cell in a specific differentiation stage. Accordingly, each threshold determines a set of (disconnected) TESs representing the distinct cell types of a specific differentiation stage, starting from toti-/multi-potent stem cells (generally characterize by a single TES including many attractors) to intermediate states, to fully differentiated cells (generally characterized by one attractor-TESs). Thus, such an emergent hierarchical structure resembles and can be compared to that of differentiation trees in real cells. Notice that the approach is general as it does not refer to any specific organism (see Fig. 1 for a simplified representation of the model).

## Implementation

CABERNET is a Java tool developed as Cytoscape’s version 3.x application; see the Availability Section for information about download and installation of the tool. CABERNET sessions are user-defined batch computations. Parameters are specified by a step-by-step wizard and various post-simulation functions are accessible directly from the CABERNET menu in the Cytoscape active window. The tool implements a wide range of simulation and analysis functions, which can be summarily listed as follows (see Table 1 for a summary of functions and parameters, and the user manual, on the plugin website, for a detailed description). Instructions on obtaining and using the code used in the paper are available on the page website. Tutorials include a package to reproduce the example application discussed in the paper (see Availability).

**Table 1:** Main functionalities and parameters of CABERNET. A schematic representation of the various functions and parameters of CABERNET is provided, as explicitly described in the Implementation Section in the main text. For a thorough explanation please refer to the user manual (see Availability)

	

### Input GRNs (generation, import and augmentation)

**[Random network generation]**CABERNET can randomly generate and simulate ensembles of GRNs with certain structural parameters such as: (*i*) number of genes; (*ii*) ingoing/outgoing GRN’s topology, e.g., *fixed*, *Erd*$\ddot {o}$*s-Rényi’s random* [47], *Barabasi-Alberts’ scale-free* (either via *preferential attachment* or *power-law* generation) [48] or *Watts-Strogatz’s small-world* [49]; (*iii*) type of regulation functions (node-specific Boolean functions). Concerning the latter, these can be set by the user or randomly generated to accomodate: (*i*)*bias-based random* functions, (*ii*)*canalyzing* [9] or (*iii*)*logical* functions - expressed in the canonical AND/OR notation. Network and simulation parameters (e.g., samples size) can be defined either via an input form or a textual file^2^.

**[Import of characterized GRNs]** GRNs that are characterized with respect to both the topology and the regulatory functions, can be loaded in CABERNET via input textual file. Limited to the network topology, CABERNET can also directly import GRNs from any public database that Cytoscape interfaces with or from any database that allow export in SBML format.

**[Augmentation of GRNs]** Any GRN loaded in the tool (see above) can be augmented by CABERNET, by randomly generating a chosen number of augmented networks, in which entities and relations are added to the input network according to user-defined structural and functional parameters. Parameters for augmentation are the same that must be defined for the random generation (see above). The resulting ensemble of augmented networks will share the input topological and functional core and will differ for the randomly generated portion.

For instance, in the Results Section, the *T-helper* cell signaling network curated from [20] (40 genes and 51 regulatory interactions), is augmented with 160 further nodes and 349 edges according to a Erdos-Rényi random topology. Assessment of the functional effect of this augmented network is also discussed.

### GRN’s dynamics simulation

Given that the space of the possible configurations of a NRBN can be dramatically large (there are 2^*N*^ possible configurations for a network with *N* nodes), CABERNET can simulate the dynamics of a network by either: (*i*) uniformly sampling the initial conditions to test (via a required user-defined parameter) or (*ii*) performing an exhaustive search (for small networks only). CABERNET allows to investigate key statistics of the emerging attractors such as, e.g., number, length, robustness and reachability. The stability of any pattern to perturbations can be assessed either via *temporary flips* (with duration of 1 step) or via *permanent* gene knock-in/knock-out.

Following the simulation, CABERNET can compute and display the threshold-dependent ATN (here called the *TES network*) for specific threshold values. Different views on such a network are available in the tool and, for instance, allow to display the genes’ configurations in a pattern, and the variation of the number of TESs alongside thresholds, as proposed in [51].

### GRN-selection constrained by differentiation scheme

One might look for a GRN giving rise to a specific differentiation tree, as done e.g. in [35]. Trees can be inputed to CABERNET in textual format or from the Cytoscape active window; CABERNET can select those NRBNs that display a differentiation tree structurally similar to the loaded one, where the measure of similarity is defined by the user. This feature is implemented as a batch process scanning among generated NRBNs. Notice that, usually, a single NRBN exhibits various emerging trees, according to the various possible combinations of thresholds thus this feature is the computationally most demanding in CABERNET.

Besides, the statistically *representative* differentiation tree(s) of each specific network, defined as the most frequent emerging tree for different (sampled) threshold combinations, provided a specific tree depth, can be computed.

### Visualization

Each computational task is tracked by a progress bar. Once the simulation of the dynamics is completed, the powerful visualization capabilities of Cytoscape can be used to analyze the topological and dynamical properties of the networks.

In particular, with CABERNET it is possible to visualize: (*i*) the NRBNs, (*ii*) the *attractor graph network*, in which all the states of the attractors and the transitions among them are displayed, (*iii*) the threshold dependent ATNs and (*iv*) the representative differentiation tree. By clicking on a specific network, it gets visualized within Cytoscape, so that it can be further analyzed.

Different network styles have been defined and can be selected: (*i*)CABERNET*network*, aimed at visualizing the properties of the NRBN: the color of each node being related to the Boolean function bias and the size of each node proportional to its degree, (*ii*)CABERNET*attractors*, for the visualization of the attractor graph network, (*iii*)CABERNET*TES*, for the visualization of the ATN and (*iv*)CABERNET*collapsed TES*, for the collapsed visualization of the ATN: in these last two styles, the edge size is proportional to the transition probability.

### Robustness analysis

Different kind of perturbations can be simulated to assess the robustness of the attractors of a network. In particular, it is possible to perform a user-defined number of (*i*) temporary (i.e., flips) or (*ii*) permanent (i.e., knock-in/knock-out) perturbations on (*i*) a chosen number of randomly selected nodes or (*ii*) specific nodes. The robustness analysis can be performed on single networks or on the whole ensemble of simulated GRNs.

Network’s stability is assessed via robustness analyses, by means of standard measures such as *avalanches* (i.e., the number of nodes whose activation pattern is different in a perturbation experiment with respect to the wild type scenario) and *sensitivity* (i.e., the number of perturbation experiments in which a certain node’s pattern is affected) [11]. The results of the analyses can be exported in csv files.

### Network analysis

CABERNET offers a wide range of network-specific statistics. These include (*i*) the distribution of the attractors’ lengths, (*ii*) the basins of attraction, (*iii*) the proportion of frozen and oscillating nodes, plus other classical network measures such as *clustering coefficient*, *network diameter* and *average path length*. All the statistics can be visualized and exported; further network measures are accessible via the network analysis tools included in Cytoscape.

### Outputs

All the networks and the relative topological, functional and dynamical properties can be exported as textual files, from both the Wizard and the Function menu. For instance, the complete topological and functional description of the networks can be exported so that it can be used in simulation environments external to Cytoscape such as, e.g., CHASTE [52], CompuCell3D [53] or the simulator described in [35] (see [54] for a recent review on multiscale models of multicellular systems). Also, it is possible to export the complete description of all the attractor states, as well as information of their basins on attraction.

### Comparison with other tools

In Table 2 the main features and functionalities of the state-of-the-art tools for the dynamical simulation and analysis of qualitative models of GRNs are compared, highlighting the similarities and differences and the specific relevance of CABERNET. In particular, CABERNET is positioned within the current landscape as a tool that do not require programming skills, by virtue of its graphical user interface and ease of usage. More precisely, CABERNET is a stand-alone software that does not require internet connection nor on-line storage of personal files (as opposed, e.g., to BooleSim [55] or Cell collective [56]). Among the main additional features of CABERNET, with respect to the competing tools (besides the other differences summarized in Table 2), is the possibility of either simulating specific regulatory architectures (as, e.g., in GINsim [57] and SQUAD [58]) or generating ensembles of GNRs, as typically done in complex systems and, specifically, in RBN research. Furthermore, the network augmentation module is uniquely present in CABERNET, as well as the GRN-driven modeling of the differentiation process and the explicit representation of biological noise, as discussed in the Background section.

**Table 2:** Software comparison. Comparison of the main features implemented in the principal tools for the qualitative simulation of the dynamics of GNRs [34, 55–58, 61–65]. Green cells and the symbol ‘V’ indicate feature that are implemented, as opposed to red cells and the symbol ‘X’. When the assignation is not neat a footnote provides further remarks

	

We conclude by noting that CABERNET is a follow-up of our previous software GESTODIFFERENT [34], an earlier GRN tool developed by our group. Novelties include, but are not limited to, the following key functionalities and simulation options: the network augmentation module, the simulation of specific regulatory architectures, the possibility of querying publicly available databases, new network topologies, functions and constraints, the robustness analysis and the network structural analysis modules, new visualization styles and new I/O functions.

### Performance evaluation and computational complexity

In order to provide a quantitive assessment of CABERNET computational performance and scalability, Table 3 presents the results of extensive simulations on GRNs with different size and complexity.

**Table 3:** Performance evaluation

	Ordered networks (*K*=2,*b*=0.3)				Critical networks (*K*=2,*b*=0.5)				Chaotic networks (*K*=3,*b*=0.3)			
Nodes	100	500	1000	5000	100	500	1000	5000	100	500	1000	5000
Avg. (sec)	10.20	53.66	101.82	603.56	9.61	52.53	109.65	746.80	13.62	73.22	150	1341.94
St. Dev. (sec)	1.07	1.76	2.45	29.40	0.40	1.74	3.82	203.43	0.48	2.18	6.91	39.58

The average computation time and standard deviation of 1000 steps of the dynamics is reported for different classes and sizes of NRBNs with random topology. Three classes are considered: *i*) ordered (i.e., average connectivity *K*=2, Boolean function bias *b*=0.3), *i* *i*) critical (i.e., *K*=2, *b*=0.5) and chaotic (i.e., *K*=3, *b*=0.5). Four different sizes are simulated: *N*=100,500,1000,5000. For each class and size, 10 different randomly generated networks are simulated starting form 1000 different initial conditions. The average computation time of the 10 (nets) X 1000 (initial conditions) is considered. Simulations were performed on a MacBook Pro with a 2.7 GHz dual-core Intel Core i7 processor with 4 MB shared L3 cache and 8 GB of RAM

We also remark that it is not possible to provide a precise upper bound for the size of the networks to simulate CABERNET, because, as it is known from RBN/NRBN literature, the dynamical behaviour of even small networks can be dramatically heterogeneous, strongly depending on the *dynamical regime*, which is defined by a series of key structural parameters (for a discussion on this topic pleas refer to, e.g., [3]). This aspect, which is particularly relevant when dealing with critical and chaotic networks, is reflected on the computation of the attractors and their stability, as well as on the optional tree-matching procedure. Accordingly, computation time cut-offs can be set to avoid excessively long simulation. Clearly, the generation of large ensemble of GRNs, as opposed to the simulation of single architectures, increases the risk of encountering a dynamically complex network. As a general comment, CABERNET can generate and simulate networks with a few thousands nodes, yet networks with higher average connectivity are more likely to display very heterogeneous behaviours.

In regard to the network augmentation function, its computational complexity corresponds with that of simple incremental approaches. Indeed, our general task is that of augmenting a network with *n* nodes and *m* edges, regardless its current topology, to one with *N*>*n* nodes and *M*>*m* edges. Thus its cost is that of adding *N*−*n* new nodes and *M*−*m* new edges; the way we select edges determines the Cabernet’s network type. No matter what, we can exploit standard generative algorithms with no computational overload^3^.

## Results

Our group has recently been focusing on the investigation of the dynamical properties of multicellular systems via multiscale simulations, with particular attention to the conditions that would favor the emergence and development of tumors. To this end, CABERNET was recently used to generate, simulate and visualize the GRNs ruling the behaviour of an intestinal crypt in CHASTE’s multiscale simulation engine, allowing to identify conditions for cancer’s emergence and crypt’s colonization [36]. In the following, we propose a further example to show some of the potential applications of CABERNET.

**[Augmentation of T-helper signaling network]** The signaling network of human T-helper cells was recently characterized with respect to both the topology and the regulatory functions. In [59] the dynamics of such network was simulated with a Boolean approach and it was shown that the attractors actually reproduce real gene activation patterns of distinctly differentiated T-helper cell types.

**Fig. 2:**
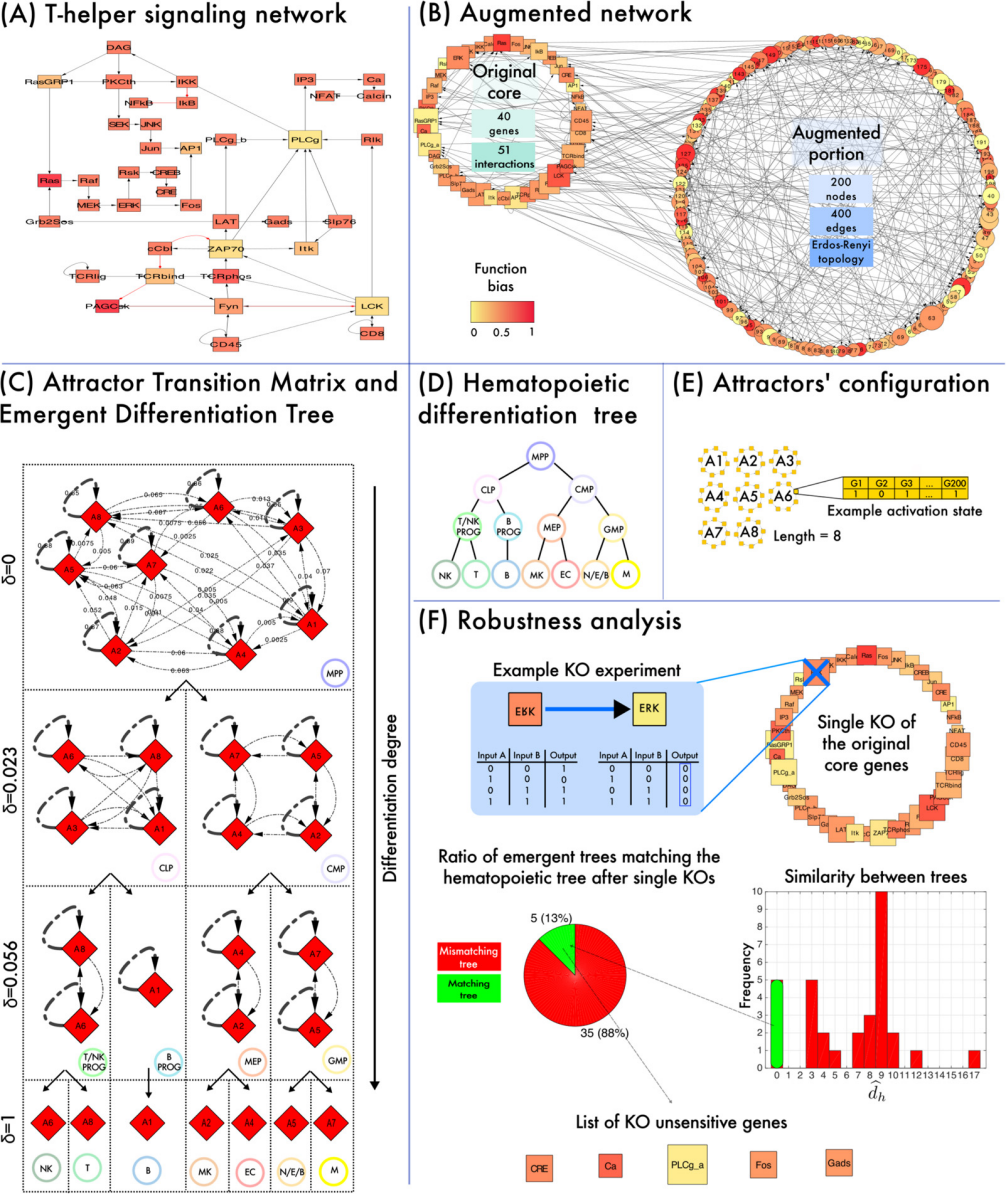
Dynamical simulation and robustness analysis of an augmented T-helper GRN with CABERNET. **a** The T-helper signaling network, mapped in [20]. Edges stand for regulatory interactions, either activating (black) or inhibiting (red). The network is composed by 40 genes and 51 interactions. **b** The augmented NRNB that displayed a differentiation tree matching the hematopoietic one. To find it, 600 NRBNs were randomly generated by augmenting the T-helper GRN in CABERNET; the augmented networks include 200 nodes (160 nodes added to the original core) and 400 edges (349 new ones, average connectivity = 2). The nodes are wired according to a random Erdos-Renyi topology, and random Boolean functions with bias = 0.5 are associated to the nodes. Only matching NRBN is shown, the original core and the augmented portion of which are highlighted. In CABERNET’s visualization the size of each node is proportional to its connectivity degree and the color-scale to the function bias. **c** The Attractor Transition Matrix of the matching NRBN is plot by CABERNET, highlighting the noise-induced transitions among attractors and the Threshold Ergodic Sets representing cell types. The progressive splitting of the TESs due to increasingly larger noise resistance-related thresholds (i.e., *δ*=0,0.023,0.056,1) is shown, stressing the perfect matching between the emergent differentiation tree and that of hematopoietic cells, from multi-potent cells to fully differentiated cell types. **d** The differentiation tree of hematopoietic cells from [60] is depicted. Notice that T-helper cell type represents one of the leaves of the tree. For the description of the acronyms please refer to the main text. **e** Configuration of the 8 attractors of the augmented network (determining the gene activation patterns). In this specific case, the length of each attractor is equal to 8. **f** Robustness analysis performed via CABERNET. Single node knockout experiments (i.e., silencing the node’s Boolean function) are performed on each node of the original core of the augmented network and the dynamics is simulated again via CABERNET. The emergent tree is then compared with that of hematopoietic cells and the distribution of the similarity measure $\widehat {d}$ (Eq. 1) is displayed, highlighting 5 genes that, when silenced, still lead to a matching emergent tree (i.e., $\widehat {d}= 0$)

The following paragraphs present an experiment of GRN’s augmentation possible in CABERNET. As far as it can be ascertained by the authors, no experiments of this sort are possible with other RBN-based tools. The final goal is to: (*i*) generate a large ensemble of random networks with the T-helper functional core, (*ii*) select only those networks in which the emergent dynamical behaviour is in accordance with the hematopoietic differentiation tree, in which the T-helper cell type is supposed to be one of the leaves, and (*iii*) eventually detect and analyze the generic properties of the selected networks, which result as *plausible* GRNs ruling the hematopoietic differentiation fate, specifically investigating their robustness (see Fig. 2).

The distinct networks differ for the augmented portion, which is randomly generated with structural parameters (i.e., Erdos-Renyi random topology, average connectivity =2, random Boolean functions with bias =0.5) that are classically used in similar studies (see, e.g., [11]). Accordingly, only certain networks will eventually display the desired emergent dynamical behavior. The underlying idea is that the *matching* networks might allow for the formulation of hypotheses on the missing portions of the relevant GRN ruling the overall hematopoiesis process. Besides, the characterization of the attractors could be matched with the real gene activation patterns driving the functioning of the various cell types^4^.

In the experiment, a large number of distinct augmented networks was generated and simulated with CABERNET: only 1 on a total of 600 augmented NRBNs actually displayed the expected dynamical behavior, i.e., the correct differentiation tree, hinting at the complexity of the tuning process driven by the evolutionary pressure that led to the topology of current GRNs^5^.

In Fig. 2 one can see the original T-helper signaling network, originally mapped in [20], and the NRBN that was selected as correct, in which the augmented portion is highlighted. Notice that, in the augmented network, both the topology and the functions of the original core are slightly different from those of the T-helper GRN, as a consequence of adding new relations linking the new and the original portions of the net. The visualization of the network is provided via CABERNET, by applying the suitable styles (see above).

In Fig. 2 one can notice that this specific network exhibits 8 distinct attractors (each one characterized by a length equal to 8 NRBN time steps). By pruning the ATN with increasingly larger thresholds, the TES at the higher level, including all the 8 attractors connected by noise-induced transitions and representing multi-/toti-potent cells, progressively splits in TESs enclosing an increasingly lower number of attractors, up to the 7 TESs at the lower level, which correspond to single attractors, when the threshold is equal to 1. This network was specifically selected as plausible because the resulting emergent differentiation tree matches that of hematopoietic cells (taken from [60], see Fig. 2)^6^.

Note that, in case augmented networks were more likely to display a matching emergent tree, one may exploit CABERNET to perform ensemble-level analyses on the matching set, aimed at the formulation of hypotheses on the generic properties of real networks.

A robustness analysis on the matching NRBN was also performed. By simulating selective single knockouts of the genes in the original T-helper core, we can assess the distinctive relevance in maintaining the correct differentiation scheme. In this example, we performed 40 single *knockout experiments* (KO), by forcing the specific Boolean function of each gene to inactivation (i.e., 0 output for any regulatory input), and we tried to match the resulting differentiation trees with that of hematopoietic cells. Remarkably, in 35 cases (88 %), the KO experiment resulted in a mismatching tree, hinting at the role of those specific genes in the interplay leading to the emergence of the hematopoietic tree. The similarity among the hematopoietic tree, *h*, and a tree *T* resulting from a KO experiment was measured as follows:

(1) $$\begin{array}{*{20}l} &\widehat{d}_{h}(T) = \sum^{l^{\ast}}_{l=0} \sum^{k^{\ast} }_{k=0}\vert n_{h}(k,l) - n_{T}(k,l)\vert \end{array} $$

where *l*^∗^ and *k*^∗^ are the maximum depth and the maximum number of a node’s children in both *h* and *T*. Function *n*_*x*_(*k,l*) returns the number of nodes at level *l* with *k* children in tree *x*; thus, this quantity measures the structural *level-by-level similarity* of two trees by assessing the number of parents with *k* children, per level. Since we focus on differentiation trees, this can be interpreted as a measure of the ability of a certain cell type, a progenitor, to differentiate in a set of distinct subtypes.

In Fig. 2 we show values of $\widehat {d}$ in our experiments; the lower the value the closer is *T* to *h*. Values of $\widehat {d}$ range around 9, with a maximum of 17 and minimum 0; in 8 cases, the value lower than 5 suggests a close similarity between the emergent and the hematopoietic tree. Besides, the dynamics turned out to be completely insensitive (i.e., $\widehat {d}= 0$) to the KO of 5 specific genes, i.e., *CRE*, *Ca*, *PLCg-a*, *Fos* and *Gads*, which, accordingly, might be not relevant in the differentiation process. Clearly, further investigations are needed to corroborate this hypothesis.

We finally remark that the generated networks could be used within any multiscale simulation frameworks, in order to investigate, e.g., the processes of homeostasis and clonal expansion, as proposed in [35, 36].

## Conclusions

In this work we introduced CABERNET - a new Cytoscape app for the generation, simulation and analysis of augmented Boolean models of gene regulatory networks - and described some of its key functionalities, as well as an example application to real GRN data.

CABERNET is the result of a long-time effort aimed at bridging different fields and disciplines, such as computer science, statistics and complex systems science, for the effective study of complex biological systems. The numerous modeling and simulation functionalities, the various effective analysis tools and the fine integration within the widely used Cytoscape framework, might settle the ground for CABERNET becoming a powerful instrument for bioinformaticians and computational biologists, especially in providing a computational support for experimentalists.

In particular, CABERNET can provide an essential tool to effectively investigate key and still partially undeciphered biological phenomena, such as, e.g., gene regulation, cell differentiation and tumorigenesis, with particular focus on the properties of dynamical gene activation patterns and their relation with biological noise.

## Availability and requirements

**Project name:**CABERNET: a Cytoscape app for the generation and the Analysis of Boolean models of gene Regulatory NETworks **Version:** 1.1 **Plugin website:**http://bimib.disco.unimib.it/index.php/CABERNET**Operating systems:** platform independent **Software requirement:** Cytoscape 3.x (http://www.cytoscape.org/) **Programming language:** Java **License:** BSD-like license (see website)

## Endnotes

^1^ We remark that one of the key advantages of employing a Boolean modeling approach lies in the possibility of including entities involved in the regulatory interplay other than genes and proteins, such as, e.g., non coding RNAs. In fact, any gene product can be considered as a Boolean entity (i.e., present/absent) interacting with other genes/products. For instance, miRNAs - non coding RNAs characterized by inhibitory functions that are able to modulate gene expression and are supposed to confer robustness against biological noise - might be represented by associating a canalyzing inhibitory function [9] to their target genes.

^2^ An extension of the software to import/export GRN models in the SBML Qual file format [50] is currently underway.

^3^ Erdos–Renyi random network are generated regardless the initial structures. For the Barabasi-Albert preferential attachment model the initial network determines the attachment probability, via the degree. For the power-law network, we sample the number of edges to assign to each node from a power law, and augment the original network accordingly. For the small word, we operate similarly. Constraint such as fixing the number of edges for a node can be set with no computational overload of this generative procedure – see the Manual for details.

^4^ Notice that the quest for an effective characterization of the relation between the functional modules of biochemical networks (i.e., sub-graphs in wider GRN models) and the phenotypic functions, in normal and aberrant cells, is nowadays central in biomedical research. This is not in contrast with the complexity of the regulation/differentiation complexity, whereas, instead, highlights the importance of the evolutionary pressure-based mechanisms that led to the specialization, modularization, hierarchical organization and plasticity of current GRNs. To this end, CABERNET can provide an effective instrument to investigate the properties of such modules, as in the presented example.

^5^ The analysis of the possible relation between the topology of the augmented portion, e.g., random, scale-free or small-world, and the likelihood of displaying the correct differentiation tree could be easily performed with the tool. We leave this interesting further application of CABERNET to future works.

^6^ The hematopoietic differentiation scheme is characterized by a multi-potent progenitor (*MPP*; antecedent hematopoietic stem cells, HSCs, are not shown in the scheme), with the potential to differentiate into two lineages, i.e., common myeloid progenitor (*CMP*) and common lymphoid progenitor (*CLP*). CMP further divide into megakaryocyte-erythroid progenitor (*MEP*) and granulocyte/monocyte progenitor (*GMP*), finally committing to mature blood cells including erythrocytes (*EC*), megakaryocyte (*MK*), monocyte (*M*) and granulocytes, i.e. neutrophils, eosinophils, basophils (*N/E/B*). Conversely, CLP further differentiate into B-cell progenitors (*B PROG*) and T-cell and natural killer cell progenitors (*T/NK PROG*), with a final commitment to mature B cells (*B*), T cells (*T*) and NK cells (*NK*) [60].
